# Light Influences How the Fungal Toxin Deoxynivalenol Affects Plant Cell Death and Defense Responses

**DOI:** 10.3390/toxins6020679

**Published:** 2014-02-20

**Authors:** Khairul I. Ansari, Siamsa M. Doyle, Joanna Kacprzyk, Mojibur R. Khan, Stephanie Walter, Josephine M. Brennan, Chanemouga Soundharam Arunachalam, Paul F. McCabe, Fiona M. Doohan

**Affiliations:** 1 Neurosurgery, Brigham and Women’s Hospital, Harvard Medical School, 4 Blackfan Circle, Boston, MA 02115, USA; E-Mail: kansari@partners.org; 2 Department of Forest Genetics and Plant Physiology, Umeå Plant Science Centre, Swedish University of Agricultural Sciences (SLU), Umeå 90 183, Sweden; E-Mail: Siamsa.Doyle@slu.se; 3 UCD Earth Institute and School of Biology and Environmental Science, College of Science, University College Dublin, Belfield, Dublin 4, Ireland; E-Mails: jkacprzyk@gmail.com (J.K.); chansundar@yahoo.com (C.S.A.); paul.mccabe@ucd.ie (P.F.M.); 4 Institute of Advanced Study in Science and Technology, Guwahati-35, India; E-Mail: mrk6@rediffmail.com; 5 Department of Integrated Pest Management, Research Centre Flakkebjerg, Forsøgsvej 1, Slagelse DK-4200, Denmark; E-Mail: stephanie-walter@gmx.net; 6 Plant Health Laboratory, Department of Agriculture and Food, Backweston, Co. Kildare, Ireland: E-Mail: josephine.brennan@agriculture.gov.ie

**Keywords:** Arabidopsis, *β*-1,3-glucanase, cell death, *Fusarium*, light, non-expressor of pathogenesis-related genes-1 (NPR1), peroxidase, phenylalanine ammonia lyase, wheat

## Abstract

The *Fusarium* mycotoxin deoxynivalenol (DON) can cause cell death in wheat (*Triticum aestivum*), but can also reduce the level of cell death caused by heat shock in Arabidopsis (*Arabidopsis thaliana*) cell cultures. We show that 10 μg mL^−1^ DON does not cause cell death in Arabidopsis cell cultures, and its ability to retard heat-induced cell death is light dependent. Under dark conditions, it actually promoted heat-induced cell death. Wheat cultivars differ in their ability to resist this toxin, and we investigated if the ability of wheat to mount defense responses was light dependent. We found no evidence that light affected the transcription of defense genes in DON-treated roots of seedlings of two wheat cultivars, namely cultivar CM82036 that is resistant to DON-induced bleaching of spikelet tissue and cultivar Remus that is not. However, DON treatment of roots led to genotype-dependent and light-enhanced defense transcript accumulation in coleoptiles. Wheat transcripts encoding a phenylalanine ammonia lyase (*PAL*) gene (previously associated with *Fusarium* resistance), non-expressor of pathogenesis-related genes-1 (*NPR1*) and a class III plant peroxidase (*POX*) were DON-upregulated in coleoptiles of wheat cultivar CM82036 but not of cultivar Remus, and DON-upregulation of these transcripts in cultivar CM82036 was light enhanced. Light and genotype-dependent differences in the DON/DON derivative content of coleoptiles were also observed. These results, coupled with previous findings regarding the effect of DON on plants, show that light either directly or indirectly influences the plant defense responses to DON.

## 1. Introduction

*Fusarium graminearum* Schwabe [teleomorph *Gibberella zeae* (Schweinitz) Petch] and *F. culmorum* (W.G. Smith) Saccardo cause diseases on the roots, stems and heads of cereal plants [1]. *Fusarium* head blight (FHB) receives significant attention because of both the yield losses and mycotoxin contamination of grain associated with this disease. *F. graminearum* and *F. culmorum* commonly produce the trichothecene mycotoxin deoxynivalenol (DON) in infected plant tissue and this toxin acts as an aggressiveness factor for the pathogen during the development of root rot and FHB disease [2,3]. DON inhibits protein synthesis and its effect on wheat (*Triticum aestivum* L.) head tissue is similar to that of FHB disease, in that it bleaches the tissue [4,5]. Wheat genotypes differ in their response to DON; resistance to DON-induced bleaching is associated with resistance to the spread of FHB disease (type II resistance to FHB), but not with resistance to *Fusarium* infection (type I resistance to FHB) [4]. 

Studies have shown that DON treatment induces defense gene transcription in wheat [5,6,7], the production of reactive oxygen species (ROS) and, thereafter, an increase in programmed cell death (PCD) [6]. Diamond *et al.* [8], however, showed that lower levels of DON (10 *vs.* 100–200 μg mL^−1^) and a DON-producing strain of *F. graminearum* did not cause cell death in *Arabidopsis thaliana* cell cultures, but they did reduce the level of cell death caused by heat shock. The opposing effects of DON on cell viability and death in Arabidopsis and wheat may be due to many factors, including the differences in DON concentrations used. We postulated that it might in part be due to light-dependent signaling. The Arabidopsis experiments were conducted using light-grown cell cultures (that contained mature chloroplasts), while the wheat experiments were conducted using seedlings. Therefore, the light exposure of cells was quite different, and it is known that DON-induced bleaching of barley tissue is light dependent [9]. The opposing effects of DON on cell death in Arabidopsis *vs.* wheat may also reflect host-dependent responses to the toxin, or a specific type of resistance to DON that is inherited in a genotype-dependent manner. DON resistance inherent to some wheat genotypes is associated with the capacity to convert DON to the less toxic DON-*3*-glucoside and co-segregated with the QTL *Fhb1* [4]. This may be in and of itself a light-dependent phenomenon, because, in Arabidopsis, a UDP glucosyltransferase (UGT) catalyzes the glucosylation of DON [10] and an analysis of Arabidopsis microarray experiments available in public repositories shows that the transcription of the encoding gene is light regulated.

The first objective of this work was to try and determine if light plays a role in how DON influences plant cell death. We show the ability of DON and DON-producing *F. graminearum* to retard cell death caused by heat shock in Arabidopsis cell cultures is light dependent, and that DON actually enhanced heat-induced cell death in dark-grown cells. The fact that the effect of DON and DON-producing *Fusarium* on cell viability was light dependent, coupled with the previously reported light-dependent bleaching of barley leaves by DON [9], indicated that light might be an important determinant of the plant response to this toxin and its producer fungi. The second objective was to determine if light influences the ability of wheat seedlings to mount defense in response to DON treatment. Using seedlings whose roots were treated with DON, we show that light does enhance defense transcript accumulation in coleoptiles and increases the DON metabolite content of coleoptiles. The extent to which light influences defense transcript accumulation and DON metabolite translocation in DON-treated seedlings, however, is wheat genotype-dependent. The implications of these results are discussed. 

## 2. Results

### 2.1. The Effect of DON on the Viability of Heat-Shocked Arabidopsis Cell Cultures Is Light Dependent

**Figure 1:**
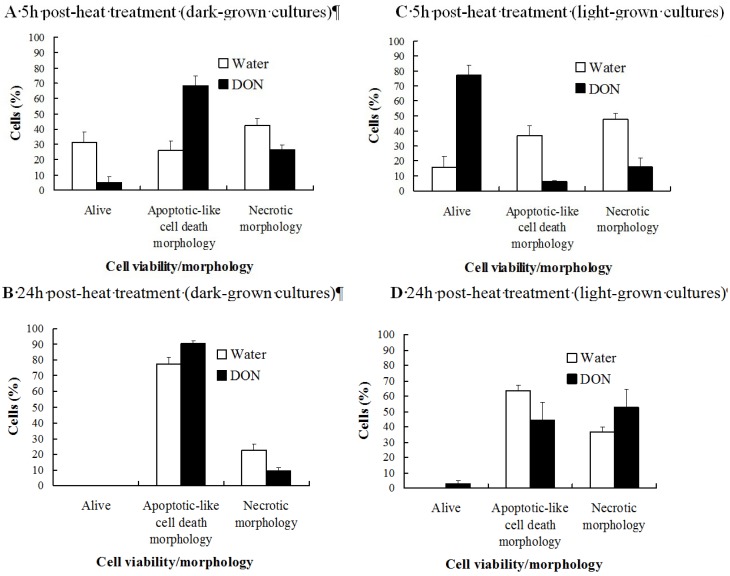
The effect of DON on the viability of heat-stressed Arabidopsis cells. Cells were cultured under dark conditions (**A** and **B**) or light conditions (**C** and **D**) and were treated with DON (10 μg mL^−1^) or water (controls) 24 h pre-heat treatment (55 °C, 10 min) and cells were examined at either (**A** and **C**) 5 h, or (**B** and **D**) 24 h post-heat treatment. Cells were treated with fluorescein diacetate and examined under phase contrast microscopy with or without UV fluorescence (490 nm) in order to determine if cells were viable, or non-viable and exhibiting either programmed cell death (PCD) or necrotic morphology. Results represent the mean percentage (+/− standard error) of cells in a given state, based on five independent experiments, and in each experiment 200 cells were scored per treatment per time point. In control, water-treated, non-heat shocked cells, ≥84% were viable and ≤14 and 3% respectively displayed PCD or necrotic morphology in dark-/light-grown cultures at the time points analyzed.

We used heat as an abiotic cell death inducer and determined whether light was necessary for DON-mediated inhibition of heat-induced cell death (a phenomenon previously discovered by Diamond *et al*., [8]). We compared light- and dark-incubated *Arabidopsis thaliana* ecotype Landsberg erecta cell cultures with respect to the effect of 10 μg mL^−1^ DON on heat-induced cell death. Light-grown cultures contained mature chloroplasts, while dark-grown cultures contained plastids, as determined by electron microscopy [11]. Cell cultures were treated with 10 μg mL^−1^ DON or water (controls) 24 h prior to heat treatment (55 °C for 10 min). Cells were treated with fluorescein diacetate and cell fluorescence and morphology (epifluorescent microscopy) were used to distinguish between viable and non-viable cells and to determine whether non-viable cells displayed apoptotic-like cell death morphology or necrotic morphology (Figure S1) [8]. Results showed that the effect of DON on cell death was light-dependent. When cells were incubated in the dark (Figure 1A, B), DON pre-treatment did not enhance the viability of heat-shocked cells. Indeed, when comparing DON and water-pretreated cells examined 5 h post-heat treatment, toxin treatment resulted in 2.6-fold higher numbers of cells exhibiting apoptotic-like cell death morphology and reduced cell viability by 5.94-fold (*P* ≤ 0.01). However, in light-grown cells harvested 5 h post-heat treatment, DON, as compared to water, reduced cell mortality (6.2 and 2.8-fold reductions, respectively in cells exhibiting apoptotic-like cell death and necrotic morphology) and enhanced the level of cell viability (by 5.0-fold; *P* ≤ 0.02; Figure 1C). Another interesting observation was that the ability of DON to inhibit heat-induced cell death in light-grown cultures was temporal. At 24 h post-treatment, the DON (relative to water) pretreatment did not influence the viability of heat-shocked cells (*P* ≥ 0.31) (Figure 1D). 

**Figure 2:**
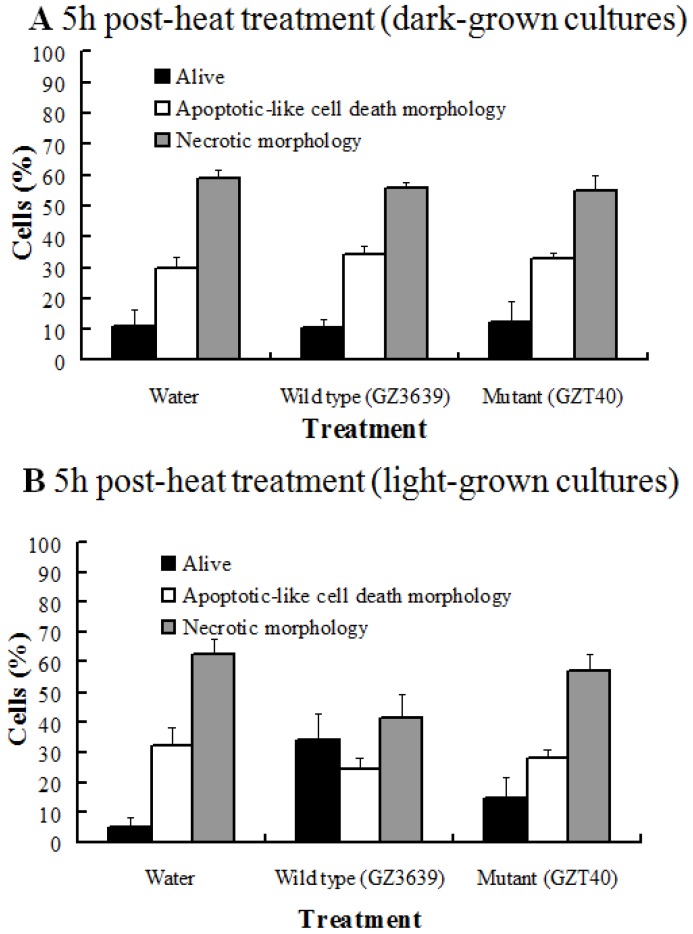
The effect of DON production by *Fusarium graminearum* on the viability of heat-stressed Arabidopsis cells. Cells were cultured under dark conditions (**A**) or light conditions (**B**) and were treated with either water, or conidia of either wild type, DON-producing *F. graminearum* (strain GZ3639) or its mutant, non-DON-producing derivative (strain GZT40). After 20 h, cells were incubated at either 23 or 55 °C for 10 min, and thereafter at 23 °C. Cells were examined at 5 h post-heat treatment. Cells were treated with fluorescein diacetate and examined under phase contrast microscopy with or without UV fluorescence (490 nm) in order to determine if cells were viable, or non-viable and exhibiting either programmed cell death (PCD) or necrotic morphology. Results represent the mean percentage (+/− standard error) of cells in a given state, based on five independent experiments, and in each experiment, a minimum of 200 cells were scored per treatment. In control, water-treated, non-heat shocked cells, ≥94% were viable and ≤4.6 and 1.4% respectively displayed PCD or necrotic morphology in dark-/light-grown cultures.

Diamond *et al.* [8] showed that, like DON, pre-treatment with DON-producing *F. graminearum* reduced the level of PCD caused by subsequent heat treatment in Arabidopsis cell cultures. We conducted similar experiments, where light- and dark-incubated Arabidopsis cell cultures were pre-treated with conidia of wild-type DON producing *F. graminearum* (strain GZ3639) or its DON-minus mutant derivative (strain GZT40), 20 h prior to heat treatment (55 °C, 10 min). Cell viability was assessed at 5 h post-heat treatment, as described above (Figure 2). For dark-grown cultures, neither the wild type nor mutant *Fusarium* significantly affected either cell viability or the morphology of dead cells (*P* ≥ 0.30) (Figure 2A). This contrasted with the DON which enhanced death under dark conditions (Figure 1). In light grown cultures, the wild-type, but not the mutant, significantly enhanced cell viability (by 2.5-fold) and reduced cell necrosis (by 1.6-fold; *P* ≤ 0.05; Figure 2B). Neither fungal strain significantly influenced the level of apoptotic-like cell death morphology observed in heat-shocked cells at this time point (*P* ≥ 0.27). Therefore we concluded that the effect of toxigenic *F. graminearum* on plant cell death is light dependent, possibly dependent on chloroplasts, and under light conditions, the ability of DON to prevent cell death caused by abiotic stress is temporal. 

### 2.2. DON Induction of Defense Gene Expression in Wheat Is Light Enhanced and Genotype Dependent

**Figure 3:**
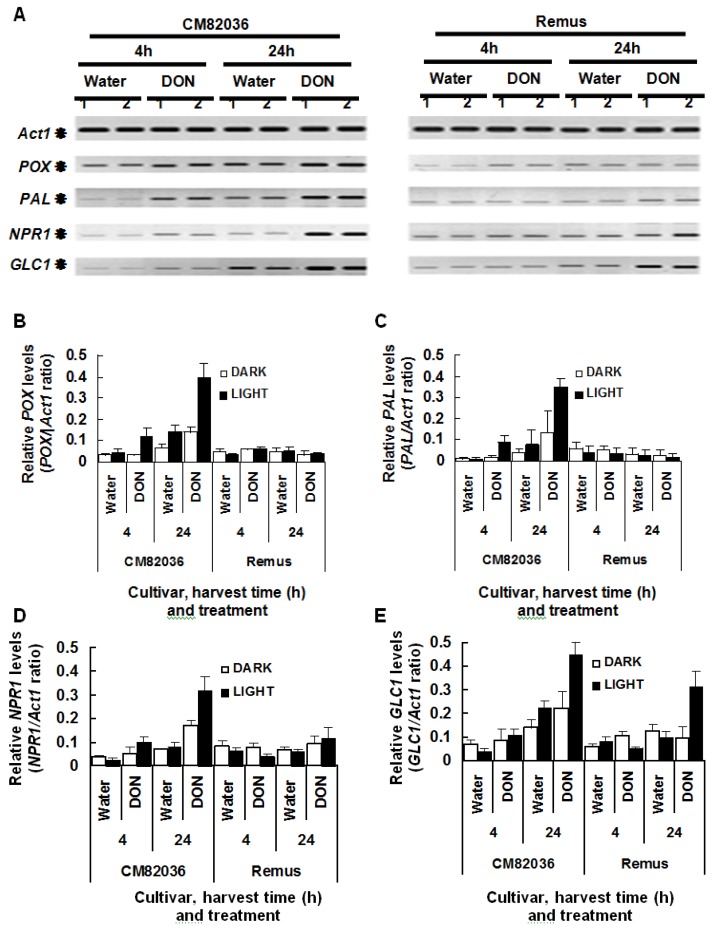
Influence of light on deoxynivalenol (DON)-induced accumulation of defense transcripts in coleoptiles of wheat cultivars CM82036 and Remus. (**A**) Visualization and (**B**–**E**) quantification of the respective *POX*, *PAL*, *NPR1* and *GLC1* transcript (relative to *Act1* transcript) levels. Results represent the mean (+/− standard error), based on two independent experiments, each including two replicates per treatment. The roots of germinated seedlings (48 h) were incubated in DON (20 mgmL^−1^) or water under light or dark conditions at 20 °C. RNA extracted from coleoptiles at either 4 or 24 h post-treatment was used for RT-PCR analyses. Gene codes: *Act1* = *actin*; *POX* = class III plant peroxidase; *PAL* = phenylalanine ammonia lyase; *NPR1* = a non-expressor of pathogenesis-related genes-1; GLC = *β*-1,3-glucanase. Arrows indicate *Act1*, *POX*, *PAL*, *NPR1* and *GLC1* RT-PCR products (270, 272, 241, 219 and 201 bp, respectively).

The second objective was to determine if light influenced the wheat response to DON. In this study, we used two wheat cultivars which differ with respect to the ability of their spikelets to resist DON; cv. CM82036 is DON-resistant, while cv. Remus is susceptible [4,5]. The response analyzed was defense gene expression, namely genes encoding *POX, PAL, NPR1* and *GLC1.* These were analyzedbecause a previous study showed that these were all DON-upregulated in wheat spikelets, and that *NPR1* transcription was more highly DON-upregulated in the toxin-treated spikelets of cv. CM82036, as compared to cv. Remus ([12]). Furthermore, the upregulation of the *PAL* gene is associated with two quantitative trait loci (QTL) that confers spikelets of cv. CM82036 with enhanced resistance to both FHB and DON [13]. In this study, the roots of seedlings grown under light or dark conditions were treated with DON and defense gene transcription in both roots and coleoptile was analyzed at 4 and 24 h post-treatment. The localized effect of DON on defense gene expression in roots of the two cultivars was not light enhanced (results not shown). In coleoptiles of cv. Remus, *GLC1* was the only transcript that was significantly DON-upregulated; this phenomenon was light-enhanced (3.1-fold higher in DON *vs.* water treated, light-grown cells observed 24 h post-treatment; *P* = 0.02) (Figure 3). In coleoptiles of dark-grown cv. CM82036 seedlings, *POX* was the only transcript significantly upregulated in response to DON treatment (2.3-fold at 24 h, *P* = 0.05). But, under light conditions, and by 24 h post-root treatment, DON had transcriptionally upregulated all four defense genes in coleoptiles of this cultivar (2.0–4.3-fold upregulation; *P* ≤ 0.02) (Figure 3). Moreover, the highest transcript levels were detected at 24 as compared to 4 h post-treatment. 

### 2.3. Both Genotype and Light Affect the Movement of DON Metabolites within Wheat Seedlings

**Figure 4:**
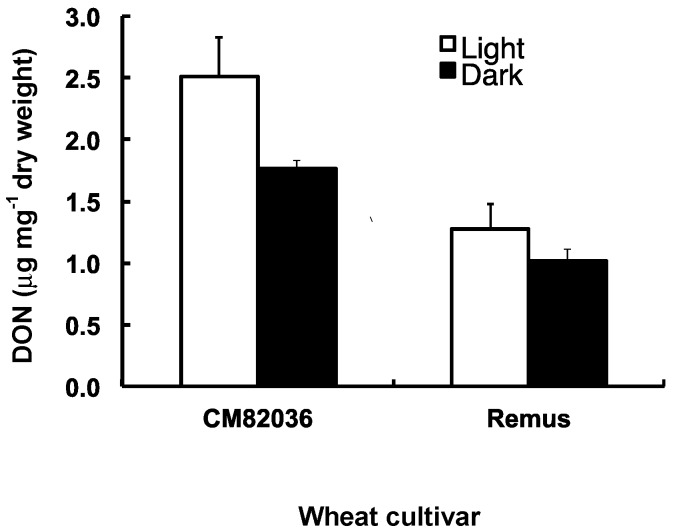
The translocation of DON metabolites within seedlings of wheat cultivars CM82036 and Remus. The roots of germinated seedlings (48 h) were incubated in DON (20 mg mL^−1^). Coleoptiles were harvested 24 h post-treatment and DON was extracted and quantified by ELISA analysis. Results represent the mean (+/− standard error), based on two independent experiments, each including two replicates per treatment.

Using an ELISA test, we determined the level of DON and DON derivatives in coleoptiles 24 h post-root treatment with toxin. The test used did not discriminate between DON and DON 3-glucoside (and thus compounds detected are described as DON metabolites). Both the incubation conditions (light/dark) and wheat genotype influenced the level of DON metabolites detected within coleoptiles (Figure 4). Coleoptiles of cv. CM82036 contained more DON metabolites than those of cv. Remus, irrespective of incubation conditions (≥1.7-fold more; *P* = 0.003) and coleoptiles of cv. CM82036, but not of cv. Remus, accumulated significantly more DON metabolites by this time when incubated under light as compared to dark conditions (1.4-fold more; *P* = 0.046). By 24 h post-DON treatment, neither light nor cultivar-dependent differences in coleoptile dry weight were observed (mean = 32–35 mg; *P* ≥ 0.20). However, coleoptiles of cv. CM82036 were more elongated than those of cv. Remus (results not shown).

## 3. Discussion

This research has established that light is an important determinant of how plants cells respond to the *Fusarium* mycotoxin DON and that wheat genotypes differ with respect to their ability to mount light-dependent defense responses to DON. Plant defense responses against pathogens, including the activation of PAL, the accumulation of SA, expression of PR proteins and the hypersensitive response, are often light dependent [14,15,16,17,18]. Light can influence defense responses via its effects on chloroplast metabolism, ROS generation and phytochrome signaling [19]. Light influences chloroplast function and several lines of evidence point to the possible role of the chloroplast as an important determinant of the plant response to DON. The light-grown Arabidopsis cells used in this study contained chloroplasts, while the dark-grown did not. DON damage of chloroplasts is a light-dependent phenomenon [9]. This could result in the accumulation of photosensitive pigments, and these can directly generate ROS in the light [20]. DON has been shown to induce ROS production in plants [6]. Excess energy produced via the photosynthetic electron transport chain [19] may contribute to the DON-induced ROS accumulation. 

It is difficult to screen for the inhibition of cell death; such a phenomenon may be interpreted as a null effect and would only be obvious in situations where cell death is induced by other factors, such as heat stress. We thus used heat to induce PCD as it provided a means to activate this conserved pathway in plants. The fact that DON and DON-production inhibits heat-induced PCD supports the previous deduction from gene expression studies. Based on microarray studies, it was deduced that ROS scavenging and the promotion of cell survival are key early defense strategies that are more effectively employed by DON-resistant as compared to susceptible wheat genotypes [7]. It would be logical for *Fusarium-*resistant plants to promote the survival, and hence the potential for defense, of cells that are being attacked by necrotrophic *Fusarium* fungi that can colonize dead plant tissue. Babaeizad *et al.* [21] showed that overexpression of a gene encoding a cell death suppressor, BAX inhibitor-1, retarded *F. graminearum* colonisation of barley seedlings. While traditionally regarded as a necrotroph, there is increasing evidence and belief that *F. graminearum* is actually a hemibiotrophic pathogen, with a short biotrophic phase preceding the necrotrophic phase of disease spread. DON suppression of death suggests that the role of DON may be to disable PCD during the initial biotrophic infection stages in plant cells, with the accumulation of higher concentrations causing PCD and facilitating nectrotrophism and disease spread. In the dark, DON enhanced the rate of apoptotic cell death. However, the fungal DON-producing strain did not do so relative to the mutant strain. There are many possible reasons for these contradictory results, including the variation in DON concentrations in toxin *versus* fungal studies and the fact that fungal factors other than DON are also very likely to affect cell death.

This study showed that defense genes were light regulated and that *NPR1, POX and PAL* were more DON responsive in seedlings of the DON-resistant cultivar CM82036, as compared to the susceptible cultivar Remus. *NPR1* and *PAL* genes have been associated with enhanced FHB resistance, but not with enhanced DON resistance. NPR1 is a central regulator of plant defense responses, including systemic acquired resistance (SAR), induced systemic resistance (ISR) and salicylic acid (SA)/jasmonic acid (JA) cross-talk [22]. An Arabidopsis *npr1-1* mutant was more susceptible to *F. graminearum* and accumulated higher concentrations of DON in buds and flowers than did wild type plants [23]. Overexpression of *Arabidopsis NPR1* (At*NPR1*) transcript in wheat conferred heritable resistance to FHB disease spread, but not initial infection [24]. These data, together with the facts that DON is associated with disease spread and that it induces the accumulation of *NPR1* transcript in wheat, suggest that the NPR1 protein is linked to Type II resistance to FHB (resistance to disease spread). In rice, overexpression of a *NPR1* homolog led to the constitutive expression of defense genes, including *POX* and *PAL* [25]. 

Steiner *et al.* [13] showed that a *PAL* transcript was *Fusarium* responsive in wheat spikelets and its responsiveness was associated with the presence of two quantitative trait loci that confer enhanced resistance to FHB, namely *Fhb1* and *Qfhs-ifa-5A.* This transcript is 100% homologous to that used herein for PCR primer design. The results of Steiner *et al.* [13] and the higher accumulation of cinnamic acid, benzoic acid, and glutamine in *F. graminearum-*infected spikelets of the FHB resistant wheat cv. Sumai-3 (a parent of cv. CM82036 that carries QTL *Fhb1*) than in spikelets of the susceptible cv. Roblin [26] provide evidence that the phenylpropanoid pathway plays a role in host defense against DON-producing *Fusaria.* QTL *Fhb1* also confers wheat with enhanced resistance to DON-induced bleaching of spikelets [4]. It is therefore likely that components of phenylpropanoid pathway play a light-dependent role in wheat resistance to DON. This is independent of DON conversion to DON-3-glucoside as Gunnaiah *et al.* [27] recently showed that *Fhb1* derived from the wheat genotype Nyubai is mainly associated with cell wall thickening due to deposition of hydroxycinnamic acid amides, phenolic glucosides and flavonoids, but not with the conversion of DON to less toxic DON-3-glucoside.

The cv. CM82036 differs from cv. Remus in its enhanced ability to convert DON to DON-3-glucoside [4]. This derivative is detected by the ELISA test [28] and, based on spikelet studies [4], it is likely to be the predominant DON metabolite in cv. CM82036, though not in cv. Remus coleoptiles. It is possible that light affects the conversion of DON to DON-3-glucoside in a genotype-dependent manner. The light-enhanced translocation in cv. CM82036 may be an indirect consequence of enhanced sugar availability for the formation of the DON-3-glucoside. Whether or not translocated DON metabolites contributed to the light-enhanced defense transcript accumulation in coleoptiles is unknown. 

## 4. Experimental Section

### 4.1. Maintenance, Growth and Treatment of Arabidopsis Cells

*Arabidopsis thaliana* (ecotype Landsberg erecta) cells were grown in liquid Murashige and Skoog (MS) media ([29,30]. Cells were sub-cultured by pipetting 10 mL of culture into 100 mL of fresh media every 7 days, and were grown on a rotary shaker at 100 rpm (5 cm rotation), a constant temperature of 23 °C, and either in darkness or at a continuous light intensity of approximately 4 μmol photons m^−2^ s^−1^. DON (Sigma, UK) was dissolved in water at a concentration of 2000 μg mL^−1^ and stored at 4 °C. Conidia of *Fusarium graminearum* strain GZ3639 and its trichothecene-minus mutant derivative (strain GZT40) [31] were produced as described previously [32] and adjusted to a concentration of 5 × 10^4^ mL^−1^ H_2_O (fresh conidia were prepared for each experiment). Arabidopsis cell samples (10 mL) were transferred to sterile 100 mL conical flasks and were treated with 0.5 mL DON 24 h prior to heat treatment or with 2 mL of conidial inoculum 20 h prior to heat treatment. Controls were treated with equivalent volumes of water. For heat treatment, cell culture flasks were placed in a shaking water bath (80 rpm) that was pre-equilibrated to either 23 or 55 °C, for 10 min. Between DON/fungal and heat treatment, and subsequent to heat treatment, samples were returned to their prior growth conditions (as above, either light or dark). Samples were morphologically analyzed at either 5 or 24 h after heat treatment. Five independent experiments compared the effect of DON and water on heat/non-heat-treated cells, and another five compared the effect of *F. graminearum* wild type, mutant and water on heat/non-heat-treated cells. Each experiment included one flask per treatment per harvest time point and 200 cells were morphologically analyzed per treatment per time point per experiment.

### 4.2. Morphological Analysis of Arabidopsis Cells

Cells were examined under a Leica DM LB microscope with an attached fluorescence lamp and camera (Leica Microsystems GmBH, Wetzlar, Germany). Cells were scored as being viable or non-viable and exhibiting either necrotic or apoptotic-like cell death morphology. The vital stain fluorescein diacetate (FDA) was used to assay for live cells [30]. When FDA is excited by light at a wavelength of 490 nm, a bright green fluorescence is observed in viable cells whose plasma membrane is intact. Cells that die by necrosis do not display the protoplast retraction associated with apoptotic-like cell death and do not fluoresce, while cells that have undergone apoptotic-like cell death show a characteristic retraction of the protoplast away from the cell wall and cannot cleave FDA [8] (Figure S1). 

### 4.3. Growth and Treatment of Wheat Seedlings

Wheat (*Triticum aestivum*) cvs. CM82036 and Remus were used in this study. Cultivar CM82036 carries a major quantitative trait locus (QTL) on the short arm of chromosome 3B that is associated with resistance to both FHB disease and DON-induced bleaching of spikelets (*Fhb1*; syn. *Qfhs.ndsu-3BS*) and it carries another QTL on chromosome 5A that is associated with FHB resistance but not with DON resistance [4,33]. Cultivar Remus is susceptible to FHB and DON-induced bleaching [4,33]. Seeds were pre-germinated in the dark at 20 °C for 48 h in Petri dishes containing filter paper moistened with 7 mL of sterile water (12 seeds per plate). Germinated seeds were then air-dried for 10 min on filter paper and carefully placed in Petri dishes containing 7 mL of either water or DON (20 mgmL^−1^ water) (12 seeds per plate) such that coleoptiles were not in contact with the treatment solution. Wheat seedlings are less sensitive to DON than Arabidopsis cell cultures ([34]), hence the reason for the higher concentration as compared to the Arabidopsis studies. Plates were incubated at 20 °C under either constant darkness or constant light (~110 mmol m^−2^ s^−1^). The roots and coleoptile were harvested at 4 or 24 h post-treatment, flash frozen in liquid N_2_ and stored at −70 °C prior to either RNA or DON extraction. Seedling experiments conducted for RNA and DON analysis each included two replica plates per treatment and each experiment was conducted twice. 

### 4.4. Quantification of DON

Freeze-dried coleoptile tissue was homogenized as previously described [5]. DON/DON derivatives was extracted from coleoptiles and quantified using the Ridascreen^®^ DON Fast immunoassay (R-Biopharm AG, Darmstadt, Germany) according to the manufacturer’s instructions. The antibodies used in this assay detect DON and DON derivatives including the less phytotoxic DON-3-glucoside [27]. Values were based on the average obtained for two replicates per sample.

### 4.5. RNA Extraction and Gene-Specific RT-PCR Analyses

Freeze-dried root or coleoptile samples were homogenized and total RNA was extracted and DNase1-treated as described by Ansari *et al.* [5]. Reverse transcription of total RNA was conducted as described by Ansari *et al.* [5], except that the primer used was oligo dT_12–18_ (Life Technologies, Paisley, UK). Both the genes of interest and wheat actin (*Act1*) were PCR-amplified (separately) using gene-specific primers (Supplemental Table S1 lists GenBank accession numbers, gene-specific primer sequences and expected product sizes); *Act1* served as a control gene that was constitutively expressed in roots, coleoptile and head tissue. RT products were diluted to 100 mL and 3 mL was PCR-amplified in a 10 mL reaction containing 1 unit of Taq DNA polymerase and 1× PCR buffer (Life Technologies, Paisley, UK), 1.5 mM MgCl_2_, 150 mM each of dATP, dGTP, dCTP and dTTP, and 100 nM each of forward and reverse transcript-specific primers. PCR reactions were conducted in a Peltier thermal cycler DNA engine (MJ Research, St. Bruno, Canada) and the programme constituted 30 cycles of 94 °C for 30 s, 60 °C for 20 s and 72 °C for 45 s, with a final extension at 72 °C for 5 min. PCR products were electrophoresed through 2% (w v^−1^) agarose gels containing 0.5 mg mL^−1^ ethidium bromide and visualized using Imagemaster VDS and Liscap software (GE Healthcare Life Sciences, Buckinghamshire, UK). 

### 4.6. Data Analysis

All data analyses were conducted using Minitab (Minitab release 13^©^, 1994 Minitab Ltd, Coventry, UK). No data set followed a normal distribution, as determined using the Normality test and none could be transformed to fit a normal distribution using the Johnson Transformation tool. Non-normally distributed data (cell viability and morphology data, gene expression data (transcript/*Act1* levels in DON relative to water-treated samples) and DON data) were analyzed using the Mann-Whitney Rank sum test (confidence level 95%, alternatives of greater than, less than or equal chosen as appropriate). 

## 5. Conclusions

We have shown that the effect of DON on the viability of abiotically stressed cells and on defense gene expression in wheat is light enhanced. Future studies should investigate the role of cell death suppression, cell survival pathways and light-regulated pathways in the resistance of wheat to *Fusarium* fungi. The combination of DON and heat stress offers a valuable means by which to unravel some of the complexity of plant programmed cell death. 

## Supplementary Files

Supplementary File 1 Supplementary Material (PDF, 327 KB)
