# Supplementary material for: Light Influences How the Fungal Toxin Deoxynivalenol Affects Plant Cell Death and Defense Responses
Source: Toxins (Basel). 2014 Feb 20;6(2):679–92. doi: 10.3390/toxins6020679 (PMC3942759; doi:10.3390/toxins6020679)

Supplementary Material

Table S1. Nucleotide sequence of defence transcript-specific primers.

Transcript	GenBank accession No.	Forward primer	Reverse primer	RT-PCR product size (bp)
**Class III plant peroxidase (*POX*)**	**EU490185**	5′-GGCTCCGGTGACAACAAC-3′	5′-AGTTGATCCTCCTGCAGTTCA-3′	272
**Phenylalanine ammonia lyase (*PAL*)**	**X99725**	5′-TCTCTTGGTCTCATCTCCTCAAG-3′	5′-GGTCGATTGTGAGCAGAAGG-3′	241
**Non-expressor of pathogenesis-related genes-1 (*NPR1*)**	**AX351127**	5′-GAGCTTGTCAGGATGCTGCT-3′	5′-CACCTTTGGTTAAAAGGGAGA-3′	219
***ß*-1,3-glucanase (*GLC1*)**	**U30323**	5′-CGGACCTGCAGAACAACA-3′	**5′-TAACTGGGGTCGCTGGAG-3′**	**201**
**Actin (*Act1*)**	**AB181991**	5′-GCCCTTGATTATGAGCAGGA-3′	**5′-GCAATTCCAGGAAACATGGTA-3′**	**270**

Figure. S1. Apototic like programmed cell death (AL-PCD) and necrotic morphology in cell suspension cultures of *Arabidopsis thaliana*. (**A**) Living cells from a seven day old suspension culture stained with FDA and viewed under white and fluorescent light; (**B**) AL-PCD cell from a seven day cell suspension culture after cell death induction at 54 °C (10 min), showing condensation of the protoplast and no FDA staining; (**C**) Necrotic cells from a seven day cell suspension culture after cell death induction at 75 °C (10 min), showing no specific cell death morphology and no FDA staining. Scale bar = 30 µm.

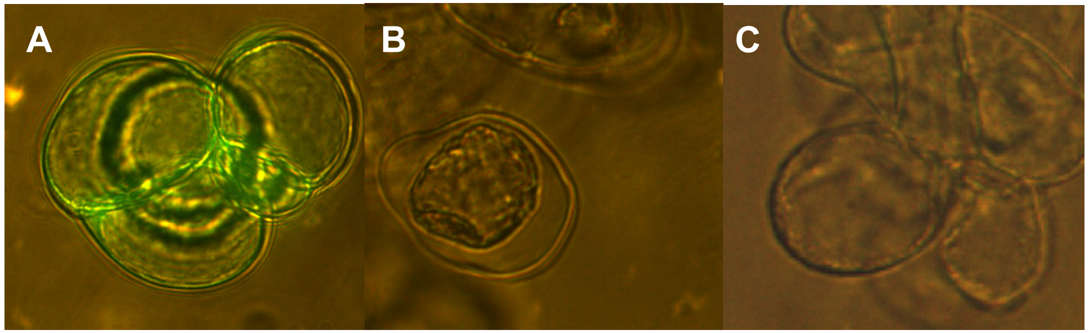

Supplement: Supplementary File 1 — Supplementary Material (PDF, 327 KB) [file toxins-06-00679-s001.pdf]
